# Comparative analysis of *Streptococcus pneumoniae* transmission in Portuguese and Finnish day-care centres

**DOI:** 10.1186/1471-2334-13-180

**Published:** 2013-04-18

**Authors:** Delphine Pessoa, Fabian Hoti, Ritva Syrjänen, Raquel Sá-Leão, Tarja Kaijalainen, M Gabriela M Gomes, Kari Auranen

**Affiliations:** 1 Instituto Gulbenkian de Ciência, Oeiras, P-2781-901, Portugal; 2 Department of Vaccination and Immune Protection, National Institute for Health and Welfare (THL), Helsinki, Finland; 3 Department of Information and Computer Science, Aalto University School of Science, Espoo, Finland; 4 Laboratory of Molecular Microbiology of Human Pathogens, Instituto de Tecnologia Química e Biológica, Universidade Nova de Lisboa (ITQB/UNL), Oeiras, Portugal; 5 Programa de Computação Científica, Fundação Oswaldo Cruz, Rio de Janeiro, 21045-900, Brazil

**Keywords:** *Streptococcus pneumoniae*, Pneumococcus, Day care, Child, Transmission, Carriage, Prevalence, Longitudinal studies, Portugal, Finland, Statistical models, Bayesian inference, Data augmentation

## Abstract

**Background:**

Day-care centre (DCC) attendees play a central role in maintaining the circulation of *Streptococcus pneumoniae* (pneumococcus) in the population. The prevalence of pneumococcal carriage is highest in DCC attendees but varies across countries and is found to be consistently lower in Finland than in Portugal. We compared key parameters underlying pneumococcal transmission in DCCs to understand which of these contributed to the observed differences in carriage prevalence.

**Methods:**

Longitudinal data about serotype-specific carriage in DCC attendees in Portugal (47 children in three rooms; mean age 2 years; range 1–3 years) and Finland (91 children in seven rooms; mean age 4 years; range 1–7 years) were analysed with a continuous-time event history model in a Bayesian framework. The monthly rates of within-room transmission, community acquisition and clearing carriage were estimated.

**Results:**

The posterior mean of within-room transmission rate was 1.05 per month (Portugal) vs. 0.63 per month (Finland). The smaller rate of clearance in Portugal (0.57 vs. 0.73 per month) is in accordance with the children being younger. The overall community rate of acquisition was larger in the Portuguese setting (0.25 vs. 0.11 per month), in agreement with that the groups belonged to a larger DCC. The model adequately predicted the observed levels of carriage prevalence and longitudinal patterns in carriage acquisition and clearance.

**Conclusions:**

The difference in prevalence of carriage (61% in Portuguese vs. 26% among Finnish DCC attendees) was assigned to the longer duration of carriage in younger attendees and a significantly higher rate of within-room transmission and community acquisition in the Portuguese setting.

## Background

*Streptococcus pneumoniae* (pneumococcus) is one of the most important bacterial causes of respiratory tract infections worldwide [1]. While it can cause serious illnesses, pneumococcus carriage in its ecological niche, the human nasopharynx, is generally asymptomatic. More than 90 serotypes have been described based on differences in the polysaccharide capsule [2]. Colonisation by pneumococcus can occur soon after birth and remains common in the first years of life [3], with virtually every child experiencing a sequence of colonisation events by alternating serotypes [4]. Previous statistical epidemiology studies have found that serotypes compete to some extent for colonisation, as carriage of a certain serotype interferes with subsequent colonisation by pneumococcus of a different serotype [5-10].

Serotype specificities in transmission and competition parameters are subject to ongoing research and debate [7,9,11,12]. Cauchemez et al. [11] concluded that there were no significant differences in rates of acquisition and clearance between vaccine and non-vaccine serotypes within school classes in France. Using the same dataset, de Cellès et al. [12] reported significant differences in the rates of acquisition estimated independently for different serotypes while assuming shared clearance rates. Erästö et al. [7] estimated serotype-specific parameters simultaneously in Bangladeshi families and concluded that, while more common serotypes in the study area were more often acquired from the community, these differences loose significance when conditioning on exposure within the family. More recently, Lipsitch et al. [9], focusing on the competitive ability of specific serotypes in Kenya, reported significant differences in susceptibility to competition, which appears positively correlated with the rate of clearance.

Studies that estimate pneumococcal transmission parameters have either used different models, statistical frameworks, parametrisations or transmission contexts, for example families, hospitals or day-care centres (DCCs). Our aim was to compare two different pneumococcal settings in Europe, one with high carriage prevalence (Portugal) and one with low carriage prevalence (Finland) by estimating pneumococcal transmission parameters using a single statistical method with data from a common context, in this case, DCCs. We apply a Bayesian data augmentation approach [6,13] to estimate acquisition and clearance rates in day-care centres in Portugal [14] and Finland [15]. We assume shared parameters among serotypes in each country and focus on the comparison between the two settings.

## Methods

### The empirical data

Two datasets were employed in this study, both of which have been described in detail elsewhere [14,15]. For the first dataset, all children from three rooms (*n*_*c*_ = 47) of a day care centre (DCC) with approximately 150 children in Lisbon, Portugal, were due to 11 scheduled nasopharyngeal samples with approximately one month sampling interval from February 1998 to February 1999. No samples were collected in July and August as the DCC was closed for the summer break. There were 16, 15 and 16 attendees in the three rooms, with mean age of 2 years (range 1.2-3.1) at the beginning of the study. Altogether, 80% of the scheduled samples were obtained, resulting in a total of 416 samples from the three DCC rooms.

Approval for the Portuguese study was obtained from the Ministry of Education and the director of the day care centre. Signed informed consent was obtained from the parents or guardians of all children.

For the second dataset, a portion of attendees (*n*_*c*_ = 61) in all seven rooms in three DCCs with a total of approximately 150 children in the Tampere region, Finland, were due to 10 scheduled nasopharyngeal samples with approximately one month sampling interval from September 2001 to May 2002. In the three DCCs, 25, 18 and 18 children were enrolled, respectively, and 90%, 82% and 99% of the samples were obtained, resulting in a total of 215, 148 and 179 samples. At the beginning of the study, the mean age of the enrolled children was 4.2 years (range 1.2-6.6).

In the Finnish study, informed consent was obtained from parents or guardians of the children. The study was conducted in compliance with the Helsinki Declaration and the ethics committee of the Pirkanmaa Hospital District (PSHP) gave a favourable opinion on the study protocol.

In both studies, calcium alginate swabs were taken from the deep nasopharynx. Pneumococcal identification and serotyping was done following routine procedures as previously described [14,15]. Only one serotype per positive sample was identified. In the Portuguese dataset, the genotype and antibiotic resistance of pneumococcal isolates were also identified but only the serotype information is used in the present study. Among the few clones that were merged into one serotype, most were segregated in different rooms and represented only few samples. In a reanalysis of samples from one of the Finnish day care centres (rooms F1 and F2), samples were incubated and cultured after an enrichment step. Pneumococci were identified from both blood and blood agar with gentamycin plates and serotyped. In addition, multiple serotypes were searched directly by the Quellung method [16].

### Transmission model

We modelled transmission dynamics of pneumococcal serotypes in children as previously described [6,13]. Children were assigned a state corresponding to their carriage status. Thus, at any given time, a child was either a non-carrier (state *s* = 0) or carrier of one of the *n*_*s*_ serotypes (state *s* ∈ {1, …, *n*_*s*_}). The rate of acquisition was taken to depend on the prevalence of pneumococci in the DCC room as well as on exposure from outside the room. We assumed that a carrier could only acquire serotypes different from the currently colonising serotype and that the rate of acquisition may be affected by current carriage. All serotypes were assumed to share the same acquisition and clearance rates.

For a non-carrying child *i*, the rate of acquisition of serotype *s* at time *t* was defined as

$$\alpha_{i}^{s}\left(t\right)=\frac{\beta C_{i}^{s}\left(t\right)}{n^{i}-1}+\kappa$$

where *n*^*i*^ is the number of attendees in the room of the child and $C_{i}^{s}\left(t\right)$ is the number of carriers of serotype *s* in his/her room just before time *t*. Parameter *β* is the rate at which one carrier transmits carriage to other children in the room. Parameter *к* is the community force of infection (per serotype), which can be interpreted as the part of acquisition rate which cannot be assigned to observed exposure within the room.

For an individual currently carrying pneumococcus, the rate of acquisition was multiplied by a competition parameter (relative rate) *ϕ*. The clearance rate within child *i* was modelled using a Weibull hazard with shape parameter *ρ* and rate parameter $\mu :\alpha_{i}^{0}\left(t\right)=\rho \mu^{\rho }{\left(t-t_{i}^{\text{acq}}\right)}^{\rho -1}$, where $t_{i}^{\text{acq}}$ is the acquisition time of the carriage episode. The shape parameter ρ of the Weibull distribution was set to value 3 to prefer carriage episodes with lengths close to the mean over very short carriage episodes. In particular, with this choice the median and mean of the Weibull distribution are approximately the same. In summary, the hazard rate for child *i* moving from state *r* to state *s* at time *t* was defined as

(1) $$\alpha_{i}^{r,s}\left(t\right)=\left\{\begin{array}{ll} \alpha_{i}^{s}\left(t\right), & \mathrm{if}\;r=0,\text{i}.\text{e}.\;\mathrm{for}\;\mathrm{acquiring}\;\mathrm{serotype}\;s\;\mathrm{when}\;\mathrm{non-carrier};\\ \varphi \alpha_{i}^{s}\left(t\right), & \mathrm{if}\;r,s>0,\text{i}.\text{e}.\;\mathrm{for}\;\mathrm{acquiring}\;\mathrm{serotype}\;s\;\mathrm{when}\;\mathrm{carrier}\;\mathrm{of}\;\mathrm{serotype}\;r;\\ \alpha_{i}^{0}\left(t\right), & \mathrm{if}\;s=0,\text{i}.\text{e}.\;\mathrm{for}\;\mathrm{clearing}\;\mathrm{carriage}\;\mathrm{of}\;\mathrm{any}\;\mathrm{serotype}\;r. \end{array}\right)$$

The Portuguese DCC data referred to children from three rooms. Due to the clear temporal separation caused by the summer break, these were analysed as 6 rooms (3 rooms before and 3 rooms after the summer break). The Finnish children corresponded to a total of 7 rooms (2, 3 and 2 rooms the three DCCs). For simplicity, we only specified exposure and transmission within the same room and considered transmission between rooms as part of the general community exposure.

Exposure from the community was assumed to remain constant during the study period. The number of serotypes (*n*_*s*_) the children were at risk of acquiring from contacts with people outside the rooms was assumed to be constant and equal to the total number of observed serotypes in the dataset (14 in Portugal, 20 in Finland). This choice affects the estimates of community acquisition rates per serotype but has negligible effect on the overall rate of community acquisition.

### Statistical methods

Adopting a Bayesian latent process approach, a likelihood-based estimation of the model parameters *θ* = {*β*, *κ*, *μ*, *ϕ*} was applied using the same algorithm and setup as Hoti et. al [6] with a new modification which handles the left censoring of the first episodes of carriage at the start of the follow-up (see below). A Markov chain Monte Carlo (MCMC) algorithm was constructed to sample from the joint posterior distribution of the model parameters and carriage histories (unobserved events) compatible with the observed data, including the carriage histories of children with completely missing data (59% of attendees in the Finnish dataset; no child had completely missing data in the rooms of the Portuguese dataset).

Given the carriage histories, i.e. the initial state of carriage and all times at which the state changes for all the children in the rooms, the complete data likelihood can be calculated. For child *i*, let $T_{i}^{r,s}$ denote the set of times the carriage status changes from state *r* to state *s* in the time interval ]*t*_min_,  *t*_max_], where *t*_min_ is one day before the first nasopharyngeal sample and *t*_max_ is the day after the last nasopharyngeal sample is taken in the dataset. Let $T_{i}=\left\{T_{i}^{r,s}:r,s=1,\ldots ,n_{s}\right\}$ be the collection of all times child *i* changes carriage status. The likelihood function of the model parameters *θ*, based on data from *n*_*c*_ individuals on the time interval ]*t*_min_,  *t*_max_] and defined by model (1), is

$$\begin{array}{c} p\left(T_{1},\ldots ,T_{n_{c}}|\theta \right)=\left(\prod_{i=1}^{n_{c}}\prod_{s=0}^{n_{s}}\prod_{r\neq s}\prod_{t\in T_{i}^{r,s}}\alpha_{i}^{r,s}\left(t\right)\right)\\ \;\times \text{exp}\left(-\sum_{i=1}^{n_{c}}\sum_{s=0}^{n_{s}}\sum_{r\neq s}\int_{t_{\text{min}}}^{t_{\text{max}}}\alpha_{i}^{r,s}\left(u\right)\;I_{i}^{r}\left(u\right)\;\text{du}\right) \end{array}$$

where $I_{i}^{r}\left(u\right)$ is the indicator function of child *i* being in state *r* just before time *u*.

The prior for each of the rate parameters (*β*, *к*, *μ*) was taken to be Normal with mean 0 and standard deviation 100, restricted on positive values, independently of the other parameters. The prior for ϕ was assumed to be Exponential with scale parameter 1/ln(2), corresponding to equal *a priori* probabilities for this parameter to be less or more than one.

In our model, the hazard of clearing carriage depends on the time of acquisition. Therefore, assuming that those found as carriers at the beginning of the follow-up had just started carrying may bias the estimation of the clearance rate as the durations of these episodes of colonisation appear shorter than they should. To correct for such left censoring of initial episodes of carriage at *t*_min_, we included the unobserved length ℓ of the carrying time preceding time *t*_min_ into the model. The preceding time ℓ was assigned an Exponential prior distribution with mean 60 days. Sampling of ℓ was done by proposing a new value from the prior distribution and then accepting or rejecting it according to the Metropolis-Hastings algorithm. Since the proposal distribution was taken to be the same as the prior distribution, the acceptance ratio depended only on the complete data likelihood ratio.

**Figure 1:**
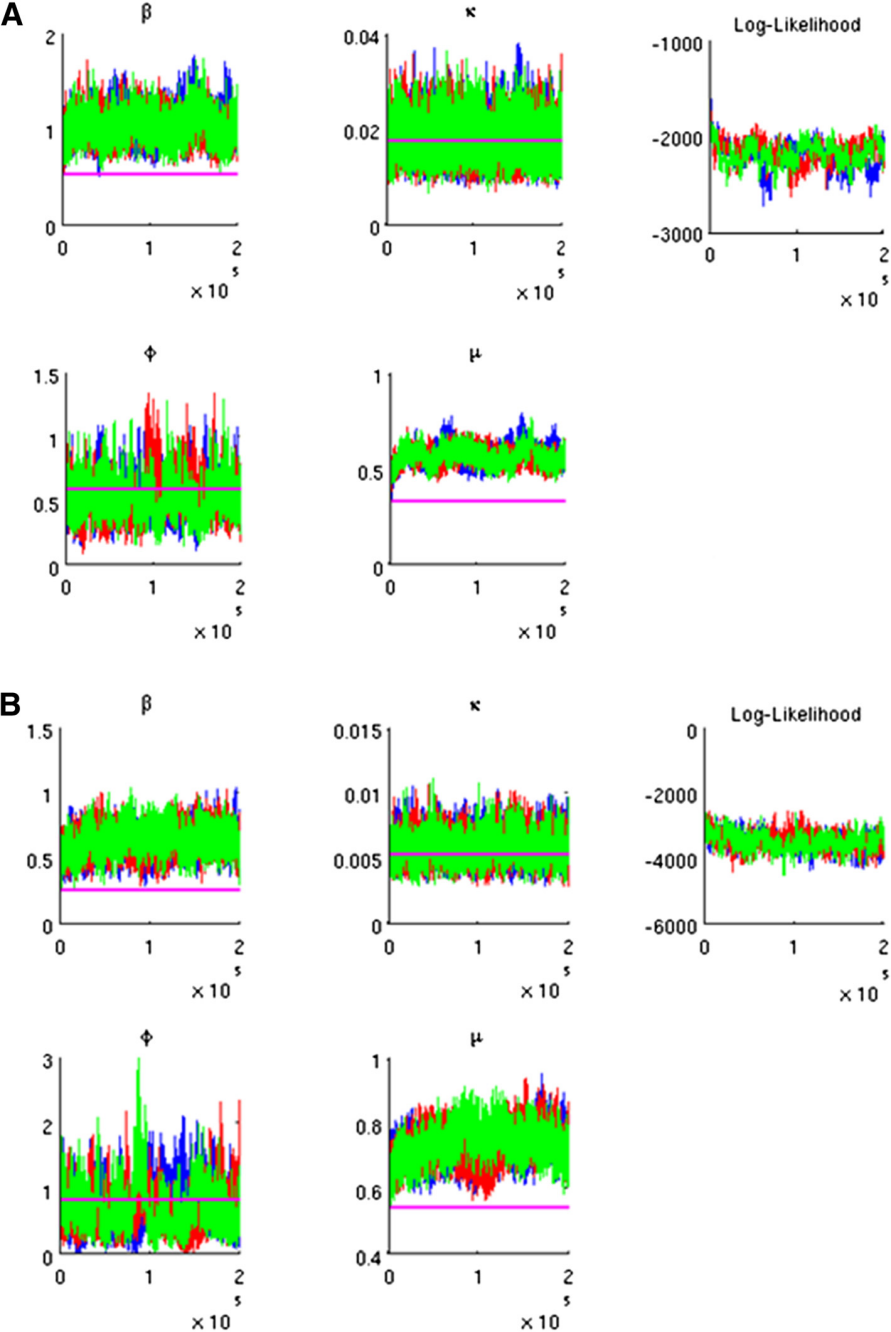
**Trace plot for the Markov chain Monte Carlo samples. A**. Portuguese data. **B**. Finnish data. Three independent Markov chains are presented. The horizontal lines present the crude estimates of the parameters. The log-likelihood at each iteration is calculated from the joint likelihood of the parameter (sample) values, based on the children’s current (augmented) histories.

The joint posterior distribution was defined as the product of the prior probabilities and the complete data likelihood (cf. [6]). Three separate MCMC chains of length 200,000 were realised for both datasets. The convergence was checked by inspection of the trace plots (Figure 1). After discarding 10,000 initial iterations from each chain, the posterior distribution was investigated from the combined 570,000 iterations, separately for the two datasets. Parameter estimates were given in terms of their posterior means and 90% credible intervals.

### Crude estimates

For comparison, crude estimates of the model parameters were calculated. These estimates were derived from a simplified model of acquisition and clearance by assuming that children acquired or cleared pneumococcus at most once between any two consecutive visits one month apart and that the events could take place only at the end of each one-month time period. Crude estimates of rates were calculated by dividing the appropriate numbers of events (acquisition or clearance) by the respective person-time at risk for the event in question, as summarised in the Appendix (see also [13]).

### Model checking

To check the model fit, data were simulated from model (1) based on a subsample of size 30,000 from the posterior of parameters *θ*. For each of the rooms, the transmission process was simulated for the total number of attendees in the room, with the initial states sampled from the overall state distribution in the respective dataset. The process was simulated for one year, following which monthly samples were taken from all children, corresponding to the number of visits in that room in the dataset. The posterior predictive distributions of the prevalence and crude estimates of the transition rates were then compared to the actually observed prevalence and crude rates. For simplicity, the transitions relating to acquisition were not adjusted for exposure within DCC rooms in this analysis.

## Results

### Data summaries and exploratory analysis

Table 1 summarises the total numbers of isolates and samples in the two datasets. The average prevalence of pneumococcal carriage was 61% (57%–64% in the 3 rooms, over the whole year) and 26% (16%–37% in the 7 rooms) in the Portuguese and Finnish datasets, respectively. Among the 254 positive samples (isolates) from the Portuguese DCC, 14 serotypes were identified, whereas 20 serotypes were identified among the 138 isolates from the Finnish DCCs. Only six serotypes were found in both datasets. The most common serotypes (with >10% of isolates) were 19F, 23F and 6B in the Portuguese dataset and 3, 18C, 9V, 15B/C and 19F in the Finnish dataset.

**Table 1:** Numbers of carriage samples and pneumococcal isolates by day-care room

	**Portugal**			**Finland**							
				**DCC1**		**DCC2**			**DCC3**		
	**P1**	**P2**	**P3**	**F1**	**F2**	**F3**	**F4**	**F5**	**F6**	**F7**	**Total:**
Number of children enrolled (/attendees)	16 (/16)	15 (/15)	16 (/16)	8 (/12)	17 (/22)	5 (/19)	5 (/21)	8 (/28)	12 (/23)	6 (/22)	108 (/194)
19F	38	24	26	0	0	3	8	3	0	0	102
23F	6	30	3	0	0	0	0	0	0	0	39
6B	6	0	23	0	1	0	1	0	0	0	31
14	1	8	13	0	0	0	1	1	0	2	26
9V	7	0	1	6	12	0	0	0	0	0	26
19A	6	7	1	4	4	0	0	0	0	0	22
3	0	0	0	0	12	2	0	0	4	3	21
10A	7	4	9	0	0	0	0	0	0	0	20
18C	0	0	0	0	0	0	0	0	18	2	20
15B/C	0	0	0	0	4	0	1	5	4	3	17
Non-typeable	4	3	8	0	0	0	0	0	0	0	15
11A	5	0	0	0	1	0	4	0	2	2	14
35F	0	0	0	0	5	0	0	2	0	0	7
16F	2	0	3	0	0	0	0	0	0	0	5
18F	2	3	0	0	0	0	0	0	0	0	5
22	0	0	0	0	0	2	3	0	0	0	5
38	0	0	0	0	3	0	0	0	0	0	3
15A	2	0	0	0	0	0	0	0	0	0	2
33	0	0	0	1	1	0	0	0	0	0	2
6A	0	0	0	0	1	0	0	1	0	0	2
10	0	0	0	1	0	0	0	0	0	0	1
16	0	0	0	0	1	0	0	0	0	0	1
18B	0	0	0	0	0	1	0	0	0	0	1
23B	0	0	1	0	0	0	0	0	0	0	1
35B	0	0	0	0	0	0	0	0	0	1	1
7	0	0	0	0	0	0	0	0	1	0	1
8	1	0	0	0	0	0	0	0	0	0	1
9N	0	0	0	0	0	0	0	0	1	0	1
Total isolates (% samples)	87 (64%)	79 (64%)	88 (57%)	12 (17%)	45 (31%)	8 (16%)	18 (37%)	12 (24%)	30 (25%)	13 (22%)	392
Negative samples	50	45	67	60	98	42	31	37	89	46	565
Total samples	137	124	155	72	143	50	49	49	119	59	957

The Portuguese day-care centre had three rooms (P1, P2, P3) and the three Finnish day-care centres (DCC1, DCC2, DCC3) had altogether 7 rooms (F1,…,F7).

**Figure 2:**
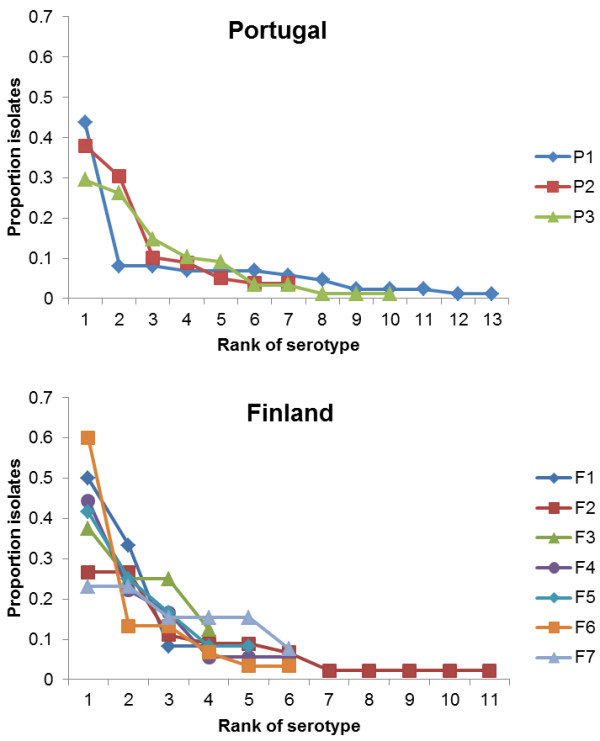
**Serotype distribution within day-care rooms.** In each room, serotypes are ranked from the most common to the least common one. There are 3 rooms (P1, P2, P3) in the Portuguese and seven rooms (F1,…,F7) in the Finnish datasets, respectively. The actual identity of each of the serotypes per room can be retrieved from Table 1.

Figure 2 shows the serotype distribution when the solates were ranked from the most common to least common one, separately for each room. The distributions are clearly skewed, with the most common serotypes in the room accounting for the majority of the isolates in that room.

**Figure 3:**
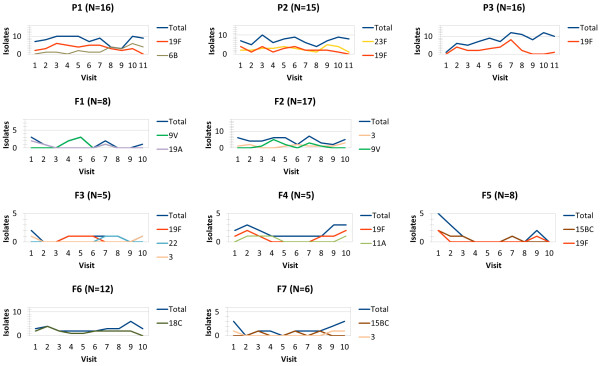
**Observed numbers of isolates for the most common serotypes by day-care room and visit.** For each room, only serotypes responsible for more than 20% of positive samples in that room are shown. The numbers of enrolled children in each room are indicated in the parentheses.

Figure 3 shows the longitudinal patterns in the observed numbers of isolates per room. Tables 2 and 3 present the observed numbers of one-month transitions in the Portuguese and Finnish datasets, respectively. Based on these data, the crude transmission rates $\hat{\beta }$ were 0.54 and 0.26 (per month) in the Portuguese and Finnish datasets, respectively. The overall crude rate of community acquisition $\left(n_{s}\;\hat{\kappa }\right)$ was 0.25 and 0.11 (per month) for Portugal and Finland, respectively. Thus, pneumococcal transmission appeared much stronger in the Portuguese dataset, both within the rooms and from the outside community. The Portuguese DCC attendees also appeared to clear colonisation slower than their Finnish counterparts, with a crude rate of clearing carriage $\hat{\mu }$ of 0.33 (per month) in Portugal and 0.54 (per month) in Finland. The crude estimates of the competition parameter $\hat{\varphi }$ were 0.80 and 1.14 based on the Portuguese and Finnish datasets, respectively.

**Table 2:** **Acquisition and clearance of *Streptococcus pneumoniae *carriage in a day-care centre in Portugal, 1998–1999**

		**Clearance**		**Acquisition in non-carriers**		**Acquisition in carriers**	
**Target serotype**	**Exposure**^**1**^	**Events**	**Person-time**^**2**^	**Events**	**Person-time**^**3**^	**Events**	**Person-time**^**4**^
19F	Yes	19	58	17	96	5	85
	No	2	2	3	19	0	20
23F	Yes	10	23	10	47	2	54
	No	1	3	2	68	1	85
6B	Yes	2	13	5	44	5	61
	No	3	7	2	71	3	84
14	Yes	2	10	3	17	6	27
	No	0	0	2	98	4	128
10A	Yes	2	11	1	27	1	44
	No	2	2	4	88	5	108
19A	Yes	3	7	1	33	0	37
	No	2	4	2	82	2	117
NT	Yes	1	5	1	44	3	52
	No	1	6	1	71	3	102
16F	Yes	0	0	0	18	0	21
	No	1	2	2	97	3	142
18F	Yes	1	1	1	4	0	7
	No	0	0	1	111	2	157
11A	Yes	0	0	0	13	1	25
	No	2	3	0	102	0	137
23B	Yes	0	0	0	6	0	4
	No	1	1	1	109	0	160
9V	Yes	0	2	0	21	1	34
	No	0	4	1	94	0	125
15A	Yes	0	0	0	4	0	7
	No	0	1	1	111	0	157
8	Yes	0	0	0	0	0	0
	No	0	0	0	115	1	165
Total:	Yes	40	130	39	374	24	458
	No	15	35	22	1236	24	1687
		55	165	78	1610	48	2145

The crude estimates of the model parameters are (cf. Appendix): the transmission rate $\hat{\beta }=\left(39-\hat{\kappa }\times 374\right)/60.18=0.54$ (per month); the community acquisition rate per serotype (in non-carrying children) $\hat{\kappa }=22/1236=0.018$ (per month); the clearance rate of carriage $\hat{\mu }=55/165=0.33$ (per month); the competition parameter $\hat{\phi }=\left(24/1687\right)/\left(22/1236\right)=0.80$. For parameter *β*, the denominator (60.18) is not directly readable from the data as presented in the table. Only samples available from two consecutive visits were considered, accounting for 67% of the scheduled pairs of consecutive visits. The three rooms were analysed as six rooms, separated by the summer break.

^1^ Exposure is here defined as the presence of at least one carrier of the target serotype in the other attendees in the room.

^2^ Person-time here is the total time, in months, spent carrying pneumococcus overall (total row) or a specific serotype (serotype rows) stratified by the presence or absence of other carriers of the specific serotype.

^3^ Person-time here is the total time, in months, spent as a non-carrier, stratified by exposure to the specific serotype. For the total row, the time is a sum of serotype-specific person-times.

^4^ As in ^2^ but for children carrying pneumococcus.

**Table 3:** **Acquisition and clearance of *Streptococcus pneumoniae *carriage in 3 day-care centres in Finland, 2001–2002**

		**Clearance**		**Acquisition in non-carrying children**		**Acquisition in carrying children**	
**Target serotype**	**Exposure**^**1**^	**Events**	**Person-time**^**2**^	**Events**	**Person-time**^**3**^	**Events**	**Person-time**^**4**^
9V	Yes	7	12	7	56	1	14
	No	3	3	6	300	1	83
18C	Yes	7	16	9	87	0	11
	No	4	4	1	269	0	81
3	Yes	1	3	6	105	1	30
	No	8	11	4	251	0	68
15BC	Yes	1	4	1	77	0	19
	No	5	12	7	279	0	77
19A	Yes	4	5	0	35	0	8
	No	3	3	2	321	0	96
19F	Yes	2	3	1	28	1	8
	No	2	8	2	328	1	93
11A	Yes	2	2	0	18	0	8
	No	0	4	1	338	5	98
14	Yes	0	0	0	12	0	5
	No	3	4	2	344	1	103
35F	Yes	0	1	1	40	0	13
	No	1	3	1	316	2	95
38	Yes	0	0	0	24	0	13
	No	2	3	2	332	0	96
22	Yes	0	0	0	20	0	0
	No	1	5	0	336	2	107
33	Yes	0	0	0	0	0	0
	No	0	0	2	356	0	112
6A	Yes	0	0	0	8	0	6
	No	1	1	1	348	0	105
6B	Yes	0	0	0	12	0	7
	No	1	2	1	344	0	103
7	Yes	0	0	0	9	0	1
	No	1	1	1	347	0	110
10	Yes	0	0	0	4	0	2
	No	1	1	0	352	0	109
18B	Yes	0	0	0	3	0	1
	No	1	1	0	353	0	110
35B	Yes	0	0	0	0	0	0
	No	0	0	1	356	0	112
9N	Yes	0	0	0	0	0	0
	No	0	0	1	356	0	112
16^5^	Yes	0	0	0	7	0	4
	No	0	0	0	349	0	108
Total:	Yes	24	46	25	545	3	150
	No	37	66	35	6575	12	1978
		61	112	60	7120	15	2128

The crude estimates of the model parameters are (cf. Appendix): the transmission rate $\hat{\beta }=\left(25-\hat{\kappa }\times 545\right)/85.26=0.26$ (per month); the community acquisition rate per serotype (in non-carrying children) $\hat{\kappa }=35/6575=0.005$ (per month); the clearance rate of carriage $\hat{\mu }=61/112=0.54$ (per month); the competition parameter $\hat{\varphi }=\left(12/1978\right)/\left(35/6575\right)=1.14$. For parameter *β*, the denominator (85.26) is not directly readable from the data as presented in the table. Only samples from two consecutive visits were considered (85% of the scheduled pairs of consecutive visits).

^1^ Exposure is here defined as the presence of at least one carrier of the target serotype in the other attendees in the room.

^2^ Person-time here is the total time, in months, spent carrying pneumococcus overall (total row) or a specific serotype (serotype rows) stratified by the presence or absence of other carriers of the specific serotype.

^3^ Person-time here is the total time, in months, spent as a non-carrier, stratified by exposure to the specific serotype. For the total row, the time is a sum of serotype-specific person-times.

^4^ As in ^2^ but for children carrying pneumococcus.

^5^ Sampled only once in a child which was missing at the next visit.

When data from rooms F1 and F2 were reanalysed with a more sensitive method of detecting pneumococcal carriage, the proportion of positive samples increased from 27% (57/215) to 29% (62/215). The proportion of multiple carriage, i.e. samples with more than one isolate was 13% (8/62).

### Adjusted analysis

**Figure 4:**
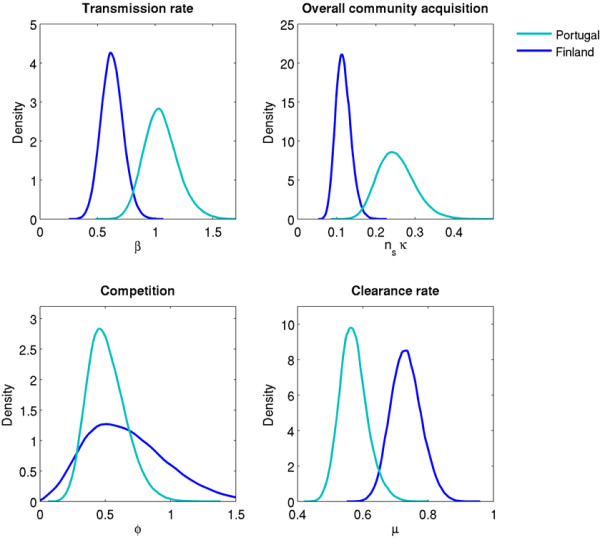
**Posterior distributions of the model parameters.** The rate parameters are presented per month.

Table 4 presents the posterior estimates of the four model parameters (see also Figure 4). The main findings are similar to those based on the crude estimates. The posterior mean transmission rate was almost double in Portugal (1.05 per month) compared to Finland (0.63 per month) with non-overlapping 90% credible intervals (0.82-1.31 vs. 0.47-0.79). The overall posterior mean community rate of acquisition was double in Portugal as compared to Finland (0.25 vs. 0.12 per month) with non-overlapping 90% credible intervals (0.18-0.34 vs. 0.09-0.15). The posterior mean of the clearance rate was lower in Portugal (0.57 per month) than in Finland (0.73 per month), but the difference was smaller than between the crude estimates. In general, the higher rates of clearance and transmission found in the full model, as compared to the crude estimates, means that the model allowed some events of acquisition and clearance to occur in between the visits even without direct observations in the samples. The posterior probability for the competition parameter (ϕ) to be less than one was 100% and 85% in the Portuguese and Finnish datasets, respectively. The posterior means were 0.51 and 0.67.

**Table 4:** Estimates of the model parameters

	**Portugal**			**Finland**		
	**Crude**	**Adjusted**		**Crude**	**Adjusted**	
		**Mean**	**(90% CI)**		**Mean**	**(90% CI)**
Transmission rate (*β*)	0.54	1.05	(0.82; 1.31)	0.26	0.63	(0.47; 0.79)
Overall community acquisition rate (*n*_*s*_*к*)	0.25	0.25	(0.18; 0.34)	0.11	0.12	(0.09; 0.15)
Clearance rate (*μ*)	0.33	0.57	(0.51; 0.65)	0.54	0.73	(0.66; 0.81)
Competition (*φ*)	0.80	0.51	(0.30; 0.77)	1.14	0.67	(0.22; 1.26)

Crude estimates and posterior means and 90% credible intervals (CI) for the four model parameters from the adjusted (full) model. The rate parameters are presented per month. The mean durations of carriage (time until immune clearance) corresponding to the crude exponential clearance rates are 3 months and 1.9 months for the Portuguese and Finnish datasets, respectively. Based on the adjusted analysis and the Weibull clearance rates, the mean durations are 1.6 months in Portugal (90% CI: 1.4-1.8 months) and 1.2 months in Finland (90% CI: 1.1-1.4 months).

### Model checking

**Figure 5:**
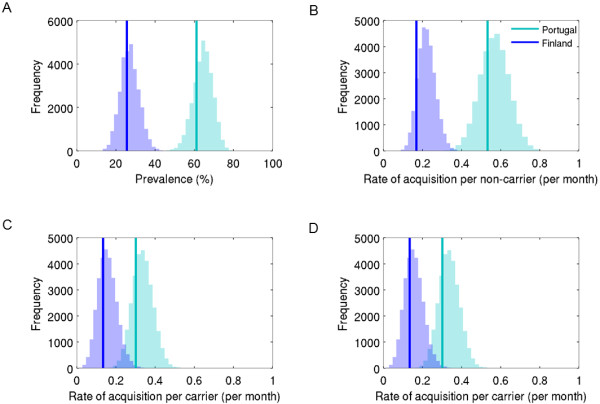
**Model checking.** The figure presents the posterior predictive distributions of carriage prevalence (panel **A**) and crude transition rates (panel **B**: acquisition in non-carriers; panel **C**: acquisition in non-carriers; panel **D**: clearance). The vertical lines show the actually observed carriage prevalence and crude transition rates. Note that the acquisition rates are not conditioned on exposure (panels **B** and **C**).

Based on the posterior sample of the model parameters, the posterior predictive mean prevalence of pneumococcal carriage was 64% in Portugal (90% predictive interval between 56% and 72%) and 27% in Finland (90% predictive interval 19%–35%) (Figure 5A). These corresponded well to the observed values (61% in Portugal and 26% in Finland). The predictive crude transition rates were also in accordance with the rates calculated directly from the data (Figures 5B-D).

## Discussion

In this article we explored what determines the prevalence of pneumococcal carriage in two European day-care settings. Parameters related to pneumococcal transmission were estimated from longitudinal studies conducted in Portugal and Finland. Differences in rates of transmission and clearance of carriage were identified as the main determinants of the observed differences in prevalence (61% in Portugal vs. 26% in Finland). Although the two studies differed in the ages of enrolled children (mean age of 2 years in Portugal vs. 4 years in Finland), this does not explain the whole extent of the prevalence difference since other studies have reported carriage levels higher than 60% among Portuguese children of age 4 years [17] and less than 40% in Finnish children of age 2 years [4]. In particular, pneumococcal transmission appears stronger in the Portuguese setting irrespective of age.

Both the rate of within-room transmission (1.05 vs. 0.63 per month) and community acquisition (0.26 vs. 0.12 per month) were twice as high in Portugal than in Finland. Cultural differences could explain the higher within-room intensity of transmission in Portugal, for example a longer time spent indoors or at the DCC. In both settings, children from the same DCC spent time together in the playground. Since we considered each of the DCC rooms separately, transmission between rooms of the same DCC would appear as community acquisition. Thus, the higher community acquisition in Portugal was probably a consequence of the Portuguese study subjects attending a larger DCC than children in any of the Finnish DCCs in this study. The credible intervals for the within-room transmission and outside acquisition were wide due to the strong (posterior) correlation between these two parameters: high within-room transmission together with low community exposure was as likely as vice-versa.

From the same dataset of Finnish DCCs, but complemented with samples from the children’s families, Hoti et al. [6] estimated a slightly lower within-DCC transmission rate of 0.53 per month and a within-family transmission rate of 0.36 per month. A study of pneumococcal transmission in U.K. families, however, estimated a higher within-family transmission rate of 1.41 per month [18]. Three previous studies have reported only small differences in transmission rates across different serotypes [7,11,12]. The transmission rates within families varied between 0.64 and 0.84 per month (Bangladesh) [13] and within DCCs between 1.04 and 1.18 [12] and between 1.38 and 1.53 [11] (France). The two French studies may have over-estimated the transmission rates, since the first [12] assumed a fixed duration of carriage of 28 days, and the second [11] assumed that carriage needs to be cleared before colonisation by another serotype.

The duration of carriage (here defined as time until immune clearance, cf. [9]) was found to be longer in Portugal (estimated mean 47 days) than in Finland (37 days). The shorter duration in older children (mean age 4 years in Finland vs. 2 years in Portugal) is in agreement with previous analyses [9,19]. Serotype 19 F [9,13] and serotype 6B [9] are typically found to be carried much longer. The fact that these serotypes were common in the Portuguese dataset but almost non-existent in the Finnish may have contributed to the estimated difference in the rates of clearance.

Similar durations of carriage were found in other studies with children under 3 years old, 48 days in Danish DCCs [8] and 51 days in English households [18]. Serotype-specific durations of carriage in Bangladeshi infants up to 1 year of age were estimated between 43 and 48 days for all serotypes except for 19 F, which was estimated to be carried on average 62 days [13]. Lipsitch et al. [9], who also estimated serotype-specific parameters, estimated the duration of carriage between 28 and 123 days. The mean duration decreased with age, 105 days in children less than 2 years old and 29 days in children between 3.5 and 5 years old.

Current carriage was found to reduce the subsequent acquisition rate by a factor of 0.5 in Portugal and 0.7 in Finland. The estimation of this competition parameter (ϕ), however, was sensitive to the choice of the prior distribution. In particular, the analysis warranted for a small prior probability for values very close to zero. In the current analysis the competition parameter was included mainly to adjust for possible confounding effects on acquisition by concurrent carriage. More data on the frequency of multiple carriage and more frequent sampling would have been necessary to learn adequately about competition [20].

The overall prevalence of pneumococcal carriage in the Finnish dataset was low, even when a more sensitive method of detecting pneumococcal carriage was applied on samples from one of the day care centres. Moreover, the proportion of multiple carriage in these samples (13%) was comparable to that in another study (10%) using the same detection method in day care children [16], although the prevalence in our study was clearly smaller (29% vs. 58%). This suggests that a low level of multiple carriage does not explain the lower prevalence and transmission rates in the Finnish data, although we did not have similar data from the Portuguese setting in this study. Moreover, it appears natural to assume that the dominant serotype is the one most likely transmitted as well as detected by sampling.

Although the prevalence of carriage and the total number of isolates were lower in Finland, the number of different serotypes was clearly larger in any of the Finnish DCCs than in the Portuguese DCC. The Portuguese children were on average younger than their Finnish counterparts (2 vs. 4 years), which could have affected the diversity of carriage: younger children carry the common paediatric types while older children and adults carry a wide variety of rarer serotypes. Cobey et al. [10] found an increase in the diversity of carriage with age. The Finnish DCCs were also more scattered in the outskirts of an urban area, such that there was probably no transmission between the DCCs and clear serotype clustering occurred, with rare serotypes being exceptionally prevalent in samples from individual DCCs [6]. The Portuguese DCC was more connected to the community and other DCCs and, as such, more mixing could occur within and across DCCs resulting in less clustering of serotypes.

Not all children in the studied DCCs were sampled. This may have caused some bias, especially in one of Finnish DCCs in which only 26% of children were sampled. In the Portuguese DCC, only three rooms were included, corresponding to 22% of the children in the DCC. Although this ensured homogeneous ages among the study subjects, the sample may not be representative of the DCC as a whole. On the other hand, one month may be too long a sampling interval, especially for the Portuguese setting, in which the prevalence and the rates of transmission and community acquisition were higher. The optimal spacing between observations has been determined for a binary model, i.e. considering only carrier and non-carriers as possible states, and varied between 1 month for very low prevalence settings and 1 week in very high prevalence settings [21]. Nevertheless, the prevalence of carriage implied by the estimated parameters was close to the observed, and may be a good representation of the transmission dynamics. It would be interesting to replicate the study reported here to a larger set of comparable longitudinal studies from different prevalence contexts or countries so as to gain a better understanding of pneumococcal transmission differences.

## Conclusion

We were successful at estimating realistic parameters for pneumococcal transmission, which were comparable among themselves and compatible with observed prevalences in two European settings (61% in Portuguese vs. 26% in Finnish day care children). The force of transmission in Portuguese children was found to be significantly higher than in the Finnish children. The community rate of acquisition, affected by the community structure, was also found higher in the Portuguese setting. The difference in carriage was explained by the higher rates of transmission and community acquisition as well as a lower rate of clearing carriage in younger DCC attendees in the Portuguese data.

## Appendix

Here we derive crude estimates for the four model parameters. Let *n*_*g*_ be the number of groups, $n_{v}^{\left(g\right)}$ the number of visits in group *g* ∈ {1, …, *n*_*g*_}, and $n_{c}^{\left(g\right)}$ the number of children in the group. For visit *v,* let $N_{v}^{r,s}$ denote the observed number of children who were in state *r* at visit *v* and in state *s* at visit *v* + 1. The total number of transitions from *r* to *s* is then the sum over all visits in all groups: $N^{r,s}=\sum_{g=1}^{n_{g}}\sum_{v=1}^{n_{v}^{\left(g\right)}-1}N_{v}^{r,s}$. Let us also consider these numbers stratified by exposure to serotype *q*: *N*^*r*,*s* (*q*+)^ and *N*^*r*,*s* (*q*−)^, where exposure is defined as presence of the serotype *q* in the room at the time of transition. The total number of samples of serotype *r* is $N^{r}=\sum_{x=0}^{n_{s}}N^{r,x}$. Stratified by exposure to serotype q, the total numbers of samples of serotype *r* are denoted by *N*^*r* (*q*+)^ and *N*^*r* (*q*−)^.

A crude estimate of the rate of clearing pneumococcus $\hat{\mu }$ is the observed number of clearance events divided by the total person-time spent carrying: $\overset{\wedge }{\mu }=\sum_{r=1}^{n_{s}}N^{r,0}/\sum_{r=1}^{n_{s}}N^{r}.$ A simple crude estimate $\hat{\kappa }$ for the community acquisition rate can be calculated from acquisition events in non-carrying children without (within-room) exposure: $\overset{\wedge }{\kappa }=\sum_{s=1}^{n_{s}}N^{0,s\;\left(s-\right)}/\sum_{s=1}^{n_{s}}N^{0\;\left(s-\right)}.$ For the transmission rate, a crude estimate $\hat{\beta }$ is derived from acquisition events in non-carrying children with exposure:

$$\overset{\wedge }{\beta }=\frac{\sum_{s=1}^{n_{s}}N^{0,s\;\left(s+\right)}-\hat{\kappa }\sum_{s=1}^{n_{s}}N^{0\;\left(s+\right)}}{\sum_{g=1}^{n_{g}}\sum_{v=1}^{n_{v}^{\left(g\right)}-1}\sum_{s=1}^{n_{s}}\frac{N_{v}^{s}}{n_{c}^{\left(g\right)}-1}N_{v}^{0\;\left(s+\right)}}.$$

This expression is adjusted for exposure from the outside community (the second term in the numerator) and from the children in the group (the denominator; cf. Erästö et al., Stat Medicine, 2012), where $N_{v}^{s}$ is the number of carriers of serotype *s* at visit *v* and $n_{c}^{\left(g\right)}$ is the number of children in the group. Finally, a crude estimate ϕ of the competition parameter can be calculated as the ratio between the rate of community acquisition in non-carriers vs. carriers, both of which are not affected by the within-room exposure. The community rate of acquisition for non-carriers is the above-mentioned $\hat{\kappa }$. For carrying children, it is

$${\hat{\kappa }}^{\text{carriers}}=\frac{\sum_{r=1}^{n_{s}}\sum_{\begin{array}{l} s=1,\\ r\neq s \end{array}}^{n_{s}}N^{r,s\left(s-\right)}}{\sum_{r=1}^{n_{s}}\sum_{\begin{array}{l} s=1,\\ r\neq s \end{array}}^{n_{s}}N^{r,s\left(s-\right)}}$$

So the estimate for the competition parameter is $\hat{\varphi }=\frac{{\hat{\kappa }}^{\text{carriers}}}{\hat{\kappa }}$.

## Competing interests

The authors declare that they have no competing interests.

## Authors’ contributions

KA, MGMG and RSL conceived the study. RS, RSL and TK provided the data. FH designed the statistical analysis. DP carried out the statistical analysis. DP and KA drafted the manuscript. All authors commented on the manuscript, contributed to writing and approved the final version of the manuscript.

## Pre-publication history

The pre-publication history for this paper can be accessed here:

http://www.biomedcentral.com/1471-2334/13/180/prepub
